# More than a chatbot: a practical framework to harness artificial intelligence across key components to boost digital therapeutics quality

**DOI:** 10.3389/fdgth.2025.1541676

**Published:** 2025-04-24

**Authors:** Amit Baumel

**Affiliations:** Department of Community Mental Health, University of Haifa, Haifa, Israel

**Keywords:** artificial intelligence, digital health intervention, digital mental health, user engagement, intervention quality, chatbot

## Abstract

The rapid advancement of Artificial Intelligence (AI)-powered large language models has highlighted the potential of AI-based chatbots to create a new era for digital therapeutics (DTx)—digital behavioral and mental health interventions. However, fully realizing AI-potential requires a clear understanding of how DTx function, what drives their effectiveness, and how AI can be integrated strategically. This paper presents a practical framework for harnessing AI to enhance the quality of DTx by dismantling them into five key components: Therapeutic Units, Decision Maker, Narrator, Supporter, and Therapist. Each represents an aspect of intervention delivery where AI can be applied. AI can personalize Therapeutic Units by dynamically adapting content to individual contexts, achieving a level of customization not possible with manual methods. An AI-enhanced Decision Maker can recommend and sequence therapeutic pathways based on real-time data and adaptive algorithms, eliminating the reliance on predefined decision trees or exhaustive logic-driven ruling. AI can also transform the Narrator by generating personalized narratives that unify intervention activities into cohesive experiences. As a Supporter, AI can mimic remotely administered human support, automating technical assistance, adherence encouragement, and clinical guidance at scale. Lastly, AI enables the creation of a Therapist to deliver real-time, interactive, and tailored therapeutic dialogues, adapting dynamically to user feedback and progress in ways that were previously impractical before. This framework provides a structured method to integrate AI-driven improvements, while also enabling to focus on a specific component during the optimization process.

## 1 Introduction

The rapid acceleration of Artificial Intelligence (AI) powered large language models (LLMs), commonly utilized through chatbots has brought considerable attention to the use of AI-based chats in digital therapeutics (DTx)—digital behavioral and mental health interventions (1). However, to meet AI potential to increase the quality of DTx we have to address how these interventions work, what makes them better or worse at reaching their aims, and how AI can be integrated within DTx based on these insights.

In this paper, the term DTx specifically refers to web-based or mobile applications that are primarily self-guided and designed to support clinically measurable mental health and behavioral changes (2, 3). Most importantly within this context, DTx constitute a distinct research field that is evaluated worldwide, focusing on user-centric design (4), the incorporation of techniques to foster behavioral and emotional change (5–8), and standardized approaches to assessing app quality (9, 10).

This potential transformation in intervention's quality builds upon the foundations laid by earlier digital health solutions, moving from static approaches of information provision to interactive and personalized therapeutic pathways (11). The progression began with websites designed for information dissemination, followed by static e-learning programs mostly providing psychoeducation. It then evolved into more interactive approaches utilizing mobile apps and web-based programs that actively support the user during the process of therapeutic change (12). During this time studies provided data showing that users poorly engage with DTx in the real world (13–15) and that there is a trial bias increasing user engagement in study settings compared to real-world use (16). As a result, more attention has been given to product design and to how content provision and mechanisms of action, as reflected in the software's functions, impact program usage and intervention effectiveness [e.g. (17–19),].

Understanding these factors is crucial, as it enables us to learn from the past and visualize the opportunities currently available for AI technologies to enhance DTX quality. Systematic reviews examining the characteristics of DTx have suggested that user adherence (20), positive behavior change (21), and program efficacy (22) can be increased by embedding a persuasive system design focused on the incorporation of behavior change techniques. Additional efforts to evaluate the quality of DTx have led to the development of eHealth program quality criteria (9, 10) and taxonomies (8), which highlight the role of user-centric and adaptive design features. Essentially, self-guided programs with high quality do not only provide evidence based content but also lean on evidence based product design. These programs deliver a targeted, dynamically tailored, and personalized pathways, and are capable of setting appropriate goals, providing reminders, monitoring and ongoing feedback, while adapting to user's context [e.g., (23)].

The interactivity and personalization offered by the most advanced interventions meeting these qualities, prior to the use of AI, faced a ceiling effect due to the reliance on manually creating logic-driven pathways. Personalization in DTx is defined here as “the capacity to monitor user context when prompted, and tailor content and response logic to each user's specific psychosocial context in real time.” The incorporation of AI into new interventions marks a critical turning point, essentially eliminating the need for manual labor in developing these pathways, while significantly enhancing the aforementioned qualities.

This paper explores the key areas and steps needed to leverage these AI capabilities to further enhance these qualities within DTx. It is worth noting that this given its nature as a Perspective article, my aim is not to provide a systematic review of the literature or an in-depth case study of one or two apps but rather to offer insights that inform future research and development.

## 2 Defining the new capabilities AI brings to DTx

**Figure 1:**
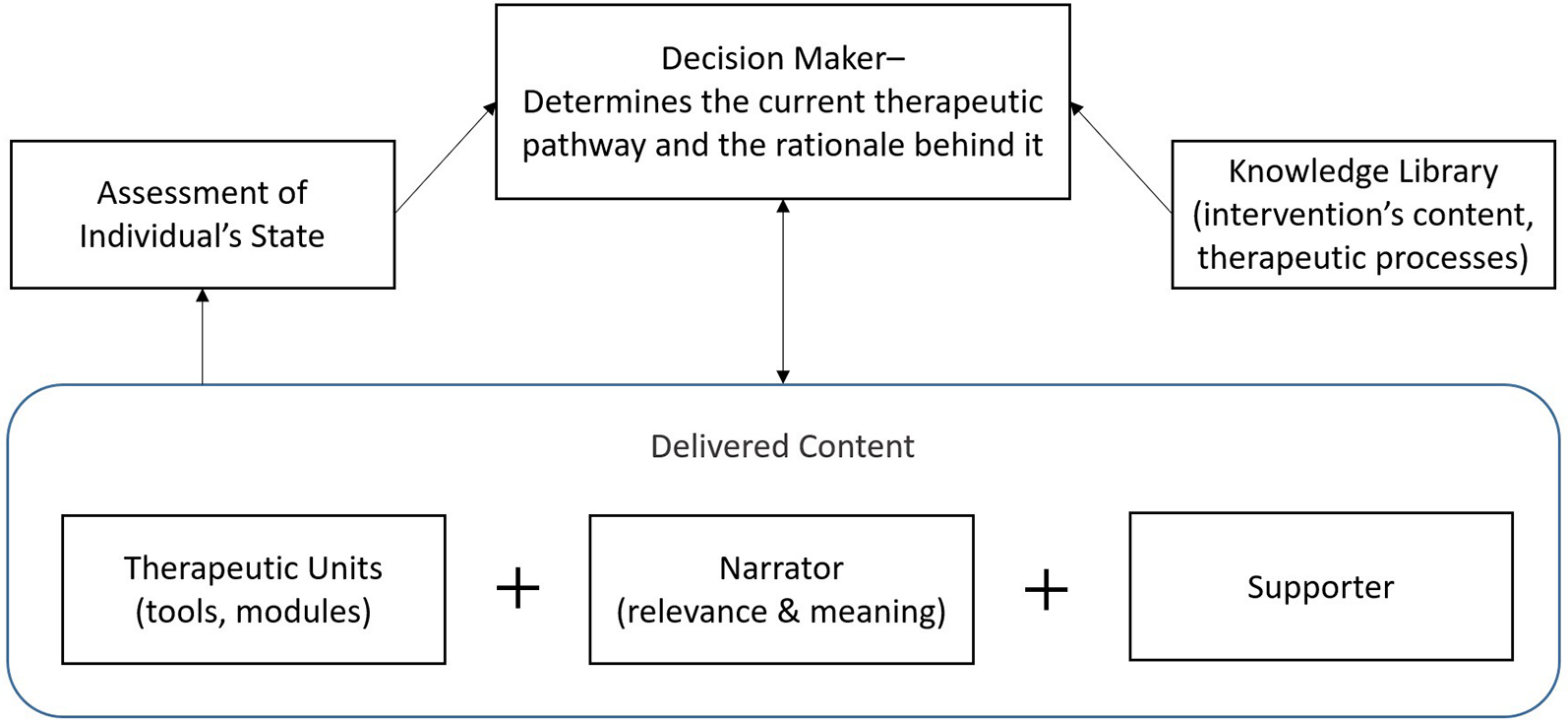
The fundamental components of digital therapeutics.

Combining LLMs with reliable knowledge on therapeutic processes, along with user context and emotion recognition, enables AI to understand inputs and to decide how to respond in a targeted, tailored, and personalized manner in real time. To help organize the discussion and demonstrate how these capabilities can be leveraged, Figure 1 illustrates the fundamental components of DTx, while Table 1 provides an explanatory overview of these components and highlights the new capabilities AI offers that could enhance their quality. In the following sections, each of these components will be discussed.

**Table 1:** An overview of DTx components and new capabilities offered by Artificial Intelligence (AI).

Component	New capabilities offered by AI	Example
Therapeutic Units. The elements designed to create a beneficial impact through their utilization.	Personalization tailored to the individual context.	Mindfulness for OCD tailored specifically for the targeted user (e.g., relationship OCD for a 30-year-old woman).
Decision Maker. Determines which units the user should engage with and provides the reasoning behind these decisions.	Endless interactions and combinations of units and reasoning without requiring comprehensive rule creation.	A person with major depression who did not find behavioral activation helpful. The Decision Maker runs an assessment and recommends the next step in the pathway, along with the reasoning behind it.
Narrator. Serves as a narrative bridge, connecting different activities to create a cohesive and consolidated user experience.	Highly personalized narratives that integrate the user's context, journey details, and system decisions.	The Narrator sends a text to a user acknowledging their success in the previous unit and explaining the rationale for the next unit while addressing the user's history.
Supporter. Aims at increasing the user's ability to effectively utilize the intervention and achieve the desired therapeutic result.	Mimicking all aspects of human support in remote conversations, including technical assistance, adherence to program guidance, and clinical support.	The Supporter identifies that a parent has not reported an improvement in positive interactions with their child, engaging the parent in dialogue to identify challenges and suggest remedies.
Therapist. Acts as a simulated mental health professional, conducting interactive sessions tailored to address the user's specific needs.	Delivering immediate, context-sensitive, and highly personalized therapeutic dialogues, adapting to the user's mood, progress, feedback, and goals.	A user with job-related anxiety interacts with the AI Therapist, which tailors the dialogue to teach relevant coping mechanisms. Over time, the AI Therapist monitors progress and fine-tunes its approach, recommending techniques like mindfulness before key meetings.

It is important to note that this framework is intended to organize the discussion in this paper regarding the potential of current AI advancements to improve DTx, rather than to comprehensively define all sub-components of DTx and how they interact with each other. For example, an essential quality aspect of a DTx is the use of triggering to engage users and to foster salience above competition (24). In the current framework, triggering is part of the Supporter role that has many facets and that can be presented in different ways (through a text message, audio call, conversation, etc.). Additionally, while specific assessments embedded within a therapeutic unit could be highly personalized based on AI, an overall assessment of individual state—based on clinically approved measurements and program usage—is not one of the components being described in this paper. The reasoning is that, much like the knowledge library, it is currently more plausible that these components will remain based on predefined data and rules curated by human experts, with limited room for AI to dynamically alter them during DTx deployment.

### 2.1 Therapeutic units

Therapeutic units are the mechanisms of action within DTx designed to achieve a specific therapeutic impact, such as mindfulness sessions for stress reduction (25), exposure technique for anxiety disorders (26, 27), or positive thinking practices for emotional well-being (28). These therapeutic units can be viewed as micro-interventions (29), evidence based Kernels (30), or active ingredients. The effective delivery of these units is dependent on technological affordance. For example, exposure techniques demand adaptability and personalization, beginning with stepped goal setting, *in vivo* guidance, monitoring, and feedback. While psychoeducation can be delivered without requiring such dynamic tailoring, exposure-based interventions are more limited in their application once the user begins practicing. For this reason, a systematic review of smartphone apps for anxiety found that exposure techniques had the largest gap between their recognition in psychotherapy protocols (85%) and their integration within apps (12%) (31). Integrating AI into these “units” enables unprecedented adaptability and personalization. Current AI tools, which also leverage diffusion models to create multimedia (32), can tailor therapeutic content to individual contexts in ways that were previously impossible.

For example, a mindfulness module designed for a 30-year-old woman with relationship-oriented Obsessive-Compulsive Disorder (OCD) can be customized by an AI system that analyzes her interaction patterns and symptoms. This allows mindfulness sessions to address her unique challenges related to relationship OCD. Theoretically, this personalized approach not only ensures that the intervention is directly relevant to her specific needs but also increases the likelihood of engagement and therapeutic success.

### 2.2 Decision maker

The Decision Maker is the function responsible for determining the most appropriate therapeutic modules and sequences to present to the user. This role involves making informed choices about which specific units to implement at various stages of the user's journey based on their evolving needs and responses. In the past, such a function did not exist, as users were streamlined through modules in a relatively one-size-fits-all format, where only the sequence or inclusion of modules could be tailored (11). Later, this function emerged in a basic form within dynamically tailored interventions, where, for example, the content of new messages became dependent on the user's past failures or successes (33, 34). Research recognize the need for dynamic, adaptive interventions that can respond to users' immediate needs rather than rely solely on static logic pathways (35).

To clarify this concept, we can use Therapeutic Persuasiveness (TP) as an example of Digital Therapeutics (DTx) quality. TP refers to the extent to which a DTx is designed to help users make beneficial changes in their lives (9). It comprises criteria such as call to action, monitoring, feedback, and data-driven decision-making. For a program to achieve a good TP quality score (four or above), it must demonstrate a high level of personalization in dynamically tailoring goals, feedback, and content. As shown in the original study assessing the quality of publicly available DTx (9), 0% of the programs received a good TP quality score. TP scores were also notably lower than content-area scores, indicating that while many programs provide solid educational material, they often struggle with real-time personalization and adaptiveness.

Recent advances indicate that machine-learning algorithms can handle real-time user input, adjusting therapeutic advice or psychoeducation based on changing symptoms (36). The incorporation of AI into this role significantly enhances its capacity to make sophisticated decisions by enabling the system to apply complex rules and algorithms without requiring exhaustive predefinitions from developers. This advancement increases the adaptiveness and personalization of care.

For instance, consider a patient with depression who did not find behavioral activation effective. The AI Decision Maker evaluates this feedback in real time, analyzes the user's ongoing psychological data, and dynamically adjusts the therapeutic strategy based on the available therapeutic units. It might recommend transitioning to cognitive behavioral therapy or mindfulness practices, providing justification for the change based on observed engagement levels and reported outcomes. This level of responsiveness not only helps maintain user engagement with the digital intervention but also ensures the treatment aligns more closely with the user's evolving needs and preferences.

### 2.3 Narrator

The Narrator serves as a guide, interpreting and explaining the therapeutic journey while making transitions between different therapeutic units understandable. This function acts as a bridge, linking various activities within the intervention into a unified experience that supports the user's meaning and expectations throughout their journey. Traditionally, the Narrator relied on predefined templates with limited variables to create personalized and context-appropriate statements (e.g., *Hi [Name], you are now moving from [prior_module_name] to [current_module_name]; your new goal is to [current_module_goal], which is highly important in your recovery journey*). This approach could be defined as “personalized communication with the user” (37), and could be utilized within triggers sent to the user (23). AI significantly enhances the Narrator's capabilities by delivering highly personalized narratives that could be deeply relevant to the user's current state and progress.

For example, an AI-powered Narrator can interact with a user managing anxiety by providing text that seamlessly introduces the next therapeutic module, explaining its selection based on the user's past responses and anticipated needs. This tailored communication helps users understand the purpose behind each step of the intervention, fostering a sense of progress and coherence throughout the therapeutic process. While systems could provide sensible narrations based on predefined variables, integrating AI enables the Narrator to adapt narratives dynamically, reflecting the user's evolving therapeutic needs and making each interaction an integral part of a well-orchestrated intervention.

Such a level of adaptability was previously only achievable with a human therapist. For instance, if a user had just experienced a traumatic event such as a car accident, a therapist would immediately adjust their narration by acknowledging the incident, exploring its psychological impact, and modifying the intervention accordingly. Similarly, AI-driven personalization can dynamically interpret user-reported events, mood changes, and contextual factors—ultimately delivering a more responsive and tailored narrative.

Importantly, the Narrator does not have to manifest as a chatbot. Narrator elements can be seamlessly integrated into various aspects of the intervention, such as triggered text messages, introductory remarks at the start of a unit, or within the user interface, depending on the system's design and interaction options. While a chatbot could enhance the sense of a working alliance, this flexibility ensures the narration can be woven into the intervention in ways that best suit the solution's architecture.

### 2.4 Supporter

The Supporter function is designed to enhance the user's ability to effectively engage with and benefit from the program, in order to achieve the desired therapeutic outcomes. Traditionally, this role has been fulfilled by a human supporter who offers a broad range of assistance to address limitations in the DTx. This support includes technical assistance, encouraging users and acknowledging their effort to maintain program adherence, and clinical guidance regarding aspects related to the DTx (38, 39). With AI technologies, it is possible to simulate human support in remote conversations. This involves not only offering technical and navigational assistance but also fostering therapeutic engagement by promoting supportive accountability (39).

For instance, if the Decision Maker detects that a parent has not reported significant improvement in interactions with their child after completing a module, the AI-powered Supporter can intervene. It might reach out to the parent to discuss challenges, offer tailored advice, or suggest alternative strategies, such as new communication techniques or additional resources to strengthen their relationship. In this way, the Supporter ensures the intervention remains responsive and tailored to the user's evolving needs, enhancing the overall effectiveness of the treatment process.

Like the Narrator, the Supporter does not have to manifest as a chatbot. It may appear as popup messages or as embedded guidance within the intervention's content. This flexibility allows the Supporter to deliver assistance in a manner that feels organic and minimally intrusive.

### 2.5 Therapist

The Therapist combines all the components discussed above into a single entity and, for this reason, does not appear in Figure 1. The Therapist is designed to act as a simulated mental health professional. This role involves conducting interactive dialogues specifically tailored to address and support the user's individual mental health needs. Historically, chatbots delivering mental health support as a therapist transitioned from rule-based systems, where users selected predefined options to receive specific answers, to pattern-matching approaches, allowing freer user input but still providing entirely predefined responses (40).

Currently, an AI Therapist could leverage a validated library containing comprehensive information about therapeutic processes of a specific clinical target, in order to reduce the risk of delivering harmful content. This Therapist may provide real-time, context-sensitive, and highly personalized therapeutic dialogues while leaning on different components (or agents), each built to take a certain role within the conversation. Subsequently, while the Therapist may lead the intervention through dialogue, it may employ other tools to gather or deliver information in the most effort optimized manner (41). For instance, assessments may be more effectively completed as surveys rather than within a dialogue, and teaching a new skill might work better through an eLearning module than via conversation.

## 3 Discussion

The components discussed rely heavily on LLMs' ability to understand text and context and to provide appropriate responses. In many respects, LLMs quality represents the upper limit of product quality and, consequently, plays a critical role in shaping the solution architecture and the presentation of such novel opportunities.

In their seminal viewpoint, Carlbring et al. argue that while machines like GPT-4 demonstrate an advanced understanding of human concepts and can even outperform humans in creating empathic text conversations, they inherently lack real emotions and personal experiences. The authors posit that these models simulate empathy rather than genuinely experience it, which might not fully substitute the need for human emotional intelligence in nuanced interactions or interpretations (e.g., understanding irony) (42). While it is genuinely questioned whether AI machines will ever possess real emotions, there are two reasons to assume that, in a relatively short period of time, AI agents could outperform humans in the quality on therapeutic conversations they conduct and the decisions they make.

The first reason is the rapid acceleration of AI model improvement in both capability, performance, and efficiency (43, 44). In the context of conversational AI, this progress suggests that models like GPT, Claude, and their successors would likely overcome current limitations in understanding subtle linguistic nuances, handling edge cases, and maintaining context over extended conversations. The second reason is that proven methodologies for reducing reliance on human support have yet to be fully implemented in AI for mental health. For instance, financial technology companies deploy innovative approaches that combine human expertise with machine learning to optimize system performance while gradually decreasing dependence on human analysts (45). For more than a decade companies, such as PayPal and Klarna, utilize fraud analysts to flag suspicious transactions, feeding these cases into machine learning algorithms that then identify patterns and automate fraud detection with increasing accuracy over time (46, 47). By applying a similar hybrid approach to mental health, involving mental health experts in the initial stages of AI system training and deployment, domain experts can establish feedback mechanisms that enable AI to grasp the subtleties of therapeutic interactions. Over time, this process could ensure that AI systems not only match but also surpass human performance.

It is critical to note, however, that even if we assume AI-led dialogues will outperform human supporters or therapists on objective conversational metrics, this does not necessarily mean that the therapeutic impact of AI agents would be greater. As biological entities with finite energy, the choices we make about where to direct our effort carry profound meaning—for ourselves and for those around us. The more effort we invest in an activity, the more meaningful it becomes to us and the more committed we are to it (41).

We apply the same understanding in our interactions with others. When a human supporter or therapist chooses to dedicate their energy to helping a patient, this act alone has therapeutic value. For the patient, knowing that another human being is investing their limited energy in their well-being fosters a unique sense of importance and connection that AI systems cannot replicate. Much like the Little Prince who learned what made his rose unique among thousands of identical roses (48), it is the time and attention a therapist “wastes” on a patient that makes the patient feel significant. This perspective does not diminish the value of AI agents but rather underscores the importance of recognizing their limitations and addressing them appropriately.

### 3.1 Future directions

There are several future directions for research based on the components presented above that warrant consideration. The first pertains to the extent to which personalization is needed outside of conversational contexts within a chatbot. For instance, while it may be assumed that more personalized content (e.g., a mindfulness exercise tailored to specific user traits) leads to higher engagement, this hypothesis has never been tested. Research is needed to examine whether AI based personalization significantly enhances therapeutic outcomes. The second concerns the optimal integration of chatbot vs. other delivery modalities. That is, it would be interesting to test to what extent and in which cases should a chatbot act as a Therapist, guiding users through other tools and monitoring their engagement, vs. serving as a Supporter to complement a standard app? Finally, it would be interesting to compare between human-supporter-led and AI-supporter-led interventions. Building on the analogy of The Little Prince, research could investigate how the perception of human effort impacts engagement and whether therapeutic pathways should integrate this understanding when incorporating AI agents.

## 4 Conclusions

This paper presents a framework for dismantling DTx into their core components, offering a structured approach to effectively integrate AI capabilities. Each component demonstrates how AI can be tailored to enhance specific aspects of intervention delivery, from personalizing therapeutic content to dynamically guiding users through their therapeutic journey. Future research should continue to explore how AI can best support and extend the impact of DTx. By building upon this component-based framework, we can pave the way for more innovative and impactful interventions, ultimately advancing the field of technology led healthcare.

## Data Availability

The original contributions presented in the study are included in the article/Supplementary Material, further inquiries can be directed to the corresponding author.
